# Total syntheses of the archazolids: an emerging class of novel anticancer drugs

**DOI:** 10.3762/bjoc.13.108

**Published:** 2017-06-07

**Authors:** Stephan Scheeff, Dirk Menche

**Affiliations:** 1 Kekulé-Institut für Organische Chemie und Biochemie, Universität Bonn, Gerhard-Domagk-Strasse 1, 53121 Bonn, Germany

**Keywords:** anticancer agent, medicinal chemistry polyketides, synthetic methodology, total synthesis

## Abstract

V-ATPase has recently emerged as a promising novel anticancer target based on extensive in vitro and in vivo studies with the archazolids, complex polyketide macrolides which present the most potent V-ATPase inhibitors known to date, rendering these macrolides important lead structures for the development of novel anticancer agents. The limited natural supply of these metabolites from their myxobacterial source renders total synthesis of vital importance for the further preclinical development. This review describes in detail the various tactics and strategies employed so far in archazolid syntheses that culminated in three total syntheses and discusses the future synthetic challenges that have to be addressed.

## Introduction

The complex structures of polyketides continues to be a great challenge for synthetic chemists and has also been a key driver for the development of new methodologies [1–9]. In many cases, total synthesis is of critical importance to enhance the supply of these often scarce metabolites and even complex polyketides have been prepared on an industrial scale [10–11]. These natural products are also valuable molecular probes for the discovery and evaluation of novel biological targets and pathways [12–13]. Vacuolar-type ATPases (V-ATPases) are heteromultimeric proton translocating proteins that are localized in a multitude of eukaryotic membranes [14–16] that have been known as key mediators in a variety of biochemical pathways. They are responsible for a variety of important cellular functions, including pH-control [17–18], membrane trafficking, protein degradation, release of neurotransmitters [18], urinary acidification [19], bone resorption [20], sperm maturation [21], cholesterol biosynthesis [22] and cytokine secretion [23]. In recent years, a key role of these multimeric enzymes also in cancer development and progression was discovered and has been increasingly unraveled. The archazolids, polyketide macrolides from the myxobacterium *Archangium gephyra*, have played a key role in these studies. They present the most potent V-ATPase inhibitors known to date with activities in the low nanomolar range [24–25], by binding to the functional transmembrane subunit c [26–27], and display highly potent growth-inhibitory activities against a range of cancer cell lines, both in vitro and in vivo [23,28–34]. In detail, archazolid inhibition of V-ATPase abrogates tumor metastasis via repression of endocytic activation [28], leads to impaired cathepsin B activation in vivo [30], modulates anoikis resistance and metastasis of cancer cells [31], overcomes trastuzumab resistance of breast cancers [32], blocks iron metabolism and thereby mediates therapeutic effects in breast cancers [33], and plays a role in tumor sensitizing in the context of the MDM2 antagonist nutlin-3a [34]. Based on these studies V-ATPases have been increasingly emerging as an extremely promising novel anticancer target [26–27,35–37] and the archazolids have become important lead structures for the development of novel anticancer agents.

**Scheme 1:**
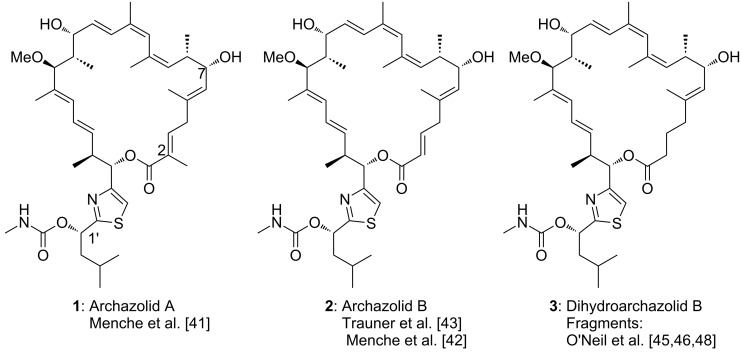
Molecular structures of the archazolids.

As shown in Scheme 1 for the most prominent representatives archazolid A (**1**) and B (**2**) [38–40], their unique architectures are characterized by a 24-membered macrolactone ring with seven alkenes, including a characteristic (*Z*,*Z*,*E*)-triene, a thiazole side chain and a characteristic sequence of eight methyl and hydroxy-bearing stereocenters.

Synthetic chemistry is of key importance to enhance the supply of these scarce polyketides to fully evaluate the biological potential and develop them as potential drug candidates. The important biological properties in combination with their limited natural supply as well as their intriguing molecular architectures and initially unknown stereochemistry, have rendered the archazolids as highly attractive synthetic targets and so far, three total syntheses have been reported by the groups of Menche and Trauner [41–43]. Furthermore several fragment syntheses as well as methodological studies to access key structural elements have been published in the last years [44–54]. Recently, the focus has been increasingly shifted towards analog development and SAR studies [49,55–58] and the group of O’Neil has been targeting dihydroarchazolid B (**3**) as a potentially equipotent structurally simplified derivative [45–46,48]. This review covers the various tactics and strategies, employed by the Menche, Trauner and O’Neil group in archazolid syntheses and discusses the challenges for the development of a scalable route.

## Review

The first total synthesis of archazolids A and B were independently developed by the Menche group and the group of Trauner in 2007 by completely independent routes. In 2009, a second total synthesis of archazolid B has been described again by the Menche group following a sequence that was related to their archazolid A synthesis.

### Menche’s retrosynthetic analysis and strategy

**Scheme 2:**
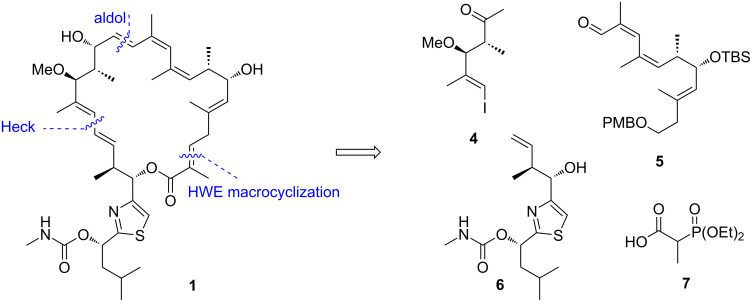
Retrosynthetic analysis of archazolid A by the Menche group.

As a prelude to initiating a synthetic campaign directed towards the archazolids the Menche group first elucidated the full stereochemistry and three dimensional conformation of the archazolids by NMR methods, molecular modelling and chemical derivatizations [59–60]. During these studies, they became aware that C2–C5 diene of acyclic analogs would be very labile towards isomerization. However, such processes would be suppressed in the macrolide natural products, presumably due to conformational factors. In contrast, the *Z*,*Z*,*E*-triene system at northern part of the target molecule (i.e., C9 to C14) was stable, also in an acyclic state, presumably due to distortion of the conjugated system due to constraints exerted by the methyl groups at C10 and C12. Based on this analysis, their synthetic plan was to first build the triene, while the C2–C5 fragment should be constructed directly before or during ring closure as shown in Scheme 2. Consequently, they first planned to connect the north-eastern fragment **5** with the propionate unit **4** by a suitable olefination strategy. Subsequently, for connecting the resulting fragment to the thiazole subunit **6** a Heck reaction was envisioned as part of studies advancing this type of Palladium-catalyzed coupling strategies in complex target synthesis [61–66]. Finally, a HWE-macrocyclization was planned which would likewise set the labile C2–C5 diene and thus concomitantly stabilize this functionality by macrocyclic constraints.

### Menche’s total synthesis of archazolid A

#### Synthesis of the north-eastern fragment

**Scheme 3:**
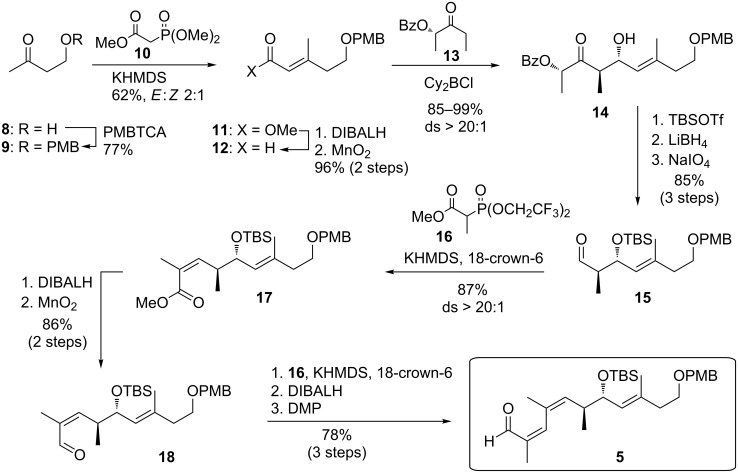
Synthesis of north-eastern fragment **5** through a Paterson *anti*-aldol addition and multiple Still–Gennari olefinations.

As shown in Scheme 3, the construction of the north-eastern fragment **5** relied on a boron-mediated *anti*-aldol reaction [67] of lactate-derived ethyl ketone **13** with aldehyde **12**, which in turn was available from aldehyde **9** by HWE olefination. This Paterson aldol reaction and related aldol reactions, which have been amply used by the Menche group [68–70], proceeded with excellent yield and selectivity. The resulting β-hydoxyketone **14** was then protected as TBS ether and converted to aldehyde **15** by reductive removal of the benzoate group and periodate cleavage. Notably, depending on the choice of protection group, deprotection and further oxidation with NaIO_4_ may be observed, a procedure that was further studied by the group [71]. The two vicinal *Z*-alkenes were then installed by two consecutive Still–Gennari olefinations [72] with aldehydes **15** and **18**. In both cases coupling with the Still–Gennari reagent **16** gave **17** and after reduction the final building block **5** was formed in high yields and selectivity. While the overall sequence towards **5** consequently required twice a two-step adaption of the oxidation state which renders this route quite lengthy, the authors argue that the robustness, facile conduction and scalability of each step was very high and made them decide to stick to this sequence as compared to likewise tested alternatives.

#### Synthesis of the north-western fragment

**Scheme 4:**
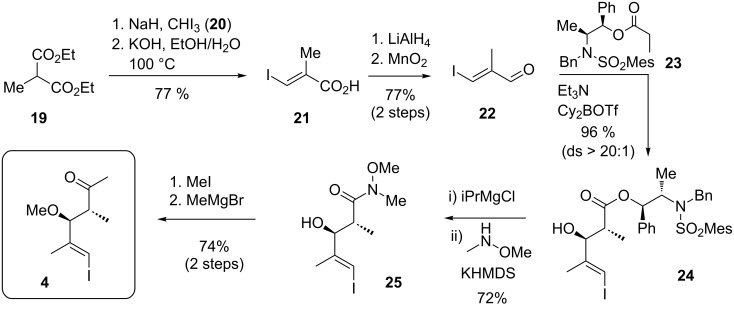
Synthesis of **4** through an Abiko–Masamune *anti*-aldol addition.

For the construction of the north-western fragment **4** the Menche group opted to first install the terminal *E*-configured vinyl iodide. While appearing to be a simple problem, quite some efforts had to be invested, before acid **21** could be efficiently obtained as shown in Scheme 4. Finally, after optimization of a reported procedure [73] the successful route employed a one-pot process involving a sodium hydride-mediated coupling of methyl malonate **19** with iodoform (**20**) followed by a stereoselective elimination and decarboxylation in situ. The corresponding aldehyde **22** was then homologated by an Abiko–Masamune *anti*-aldol addition [74] with ephedrine-derived ester **23**, which proceeded with excellent yield and stereoselectivity. However, the subsequent removal of the sterically hindered chiral auxiliary proved demanding. The Menche group realized that a transformation to a Weinreb amide may be realized in an effective manner by an in situ activation of **24** with iPrMgCl [75], followed by a methylation of the free hydroxy group and introduction of the methyl ketone. This procedure proved superior to an original sequence involving a reductive cleavage of the auxiliary.

#### Synthesis of the southern fragment

**Scheme 5:**
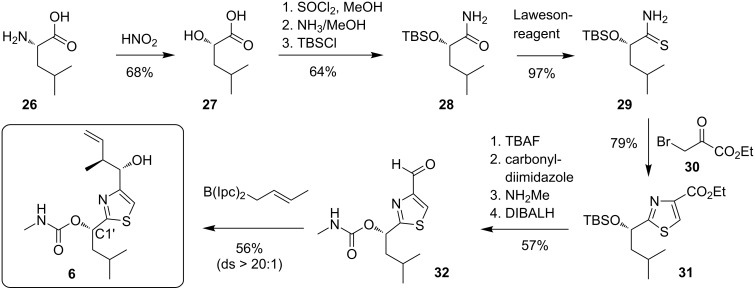
Thiazol construction and synthesis of the southern fragment **6**.

The same southern fragment **6** was independently chosen by the Menche group in their total synthesis of archazolid A and by the Trauner group in their total synthesis of archazolid B. Both groups also pursued identical routes to this subunit. As shown in Scheme 5, this sequence started from L-leucine (**26**) which was first converted with nitrous acid to the hydroxy acid **27**, which proceeds with retention of the configuration due to intermediate lactone formation after generation of the diazonium-intermediate [76]. After conversion to amide **28** and thioamide **29** the thiazole **31** was obtained by condensation with bromoester **30**. The carbamate was then introduced by activation of the deprotected hydroxy group with carbonyldiimidazole and treatment with methylamine, before the ester was selectively reduced to the aldehyde **32** with DIBALH. Finally, a Brown crotylation [77] set the two vicinal stereogenic centers of **6** with high stereoselectivity. In total this route enabled an efficient and reliable access to this key fragment. However, one drawback of this sequence was a tendency of epimerisation at C1’ during thiazole formation on large scale, requiring an oxidation–reduction sequence (90%) in this case.

#### Completion of the total synthesis

**Scheme 6:**
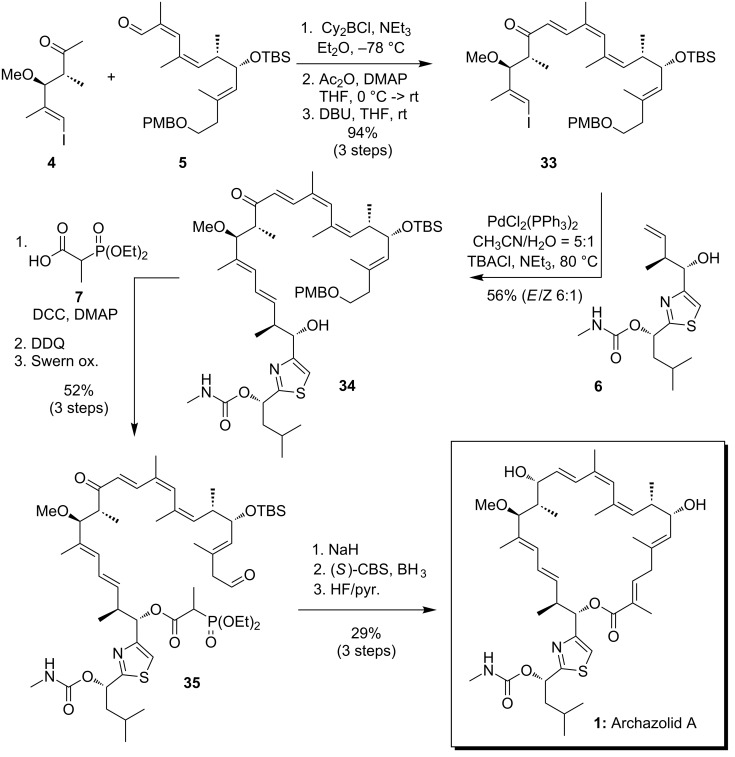
Completion of the total synthesis of archazolid A.

After evaluation of several strategies, the assembly of the two northern fragments **4** and **5** could be realized by an aldol condensation, involving a boron-mediated aldol coupling, acetate protection of the newly generated hydroxy group and DBU-mediated elimination. This three-step sequence proceeded as shown in Scheme 6 with excellent yields (94%) giving the triene **33** as a single diastereomer, which demonstrates the usefulness of aldol condensations in complex target synthesis, also on highly elaborate substrates. Considerable efforts were invested before the challenging Heck coupling with the thiazole fragment **6** could be effected with useful selectivities. Besides the desired *E*,*E*-diene **34** formation of other double bond isomers both in the southern and northern part of the molecule could not be suppressed and required a tedious HPLC separation at this stage. After attachment of the phosphonate **7**, aldehyde **35** was obtained by removal of the PMB group and oxidation of the primary alcohol. The moderate yields of this sequence are mainly due to side reactions in the deprotection step. The Menche group then had considerable difficulties in closing the macrocyclic ring using an HWE reaction. Finally, the macrocylization could be realised by using NaH as a strong non-nucleophilic base. It proved essential to perform this reaction in the presence of molecular sieves to remove any traces of water which were shown to lead to unfavourable isomerization pathways. Finally, an oxazaborolidine-assisted borane reduction [78] set the stereogenic center at C15 and global deprotection gave synthetic archazolid A, which proved to be fully identical with a natural sample, thus unambiguously confirming the stereochemistry of this macrolide [59].

### Menche’s total synthesis of archazolid B

**Scheme 7:**
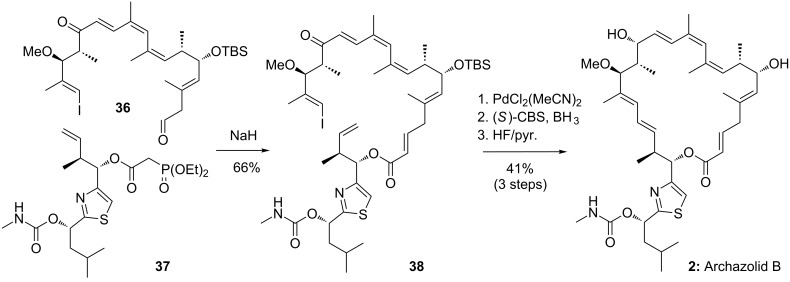
Synthesis of archazolid B (**2**) by a ring closing Heck reaction of **38**.

One the methodological incentives of the synthetic campaign of the Menche group directed towards the archazolids were the further development and application of the Heck reaction in complex target synthesis. Accordingly, they applied a Heck macrocyclization strategy for the total synthesis of archazolid B. As shown in Scheme 7, this strategy could be successfully implemented and the macrocyclic core of the target compound was obtained in useful yields by coupling of **38**, which in turn was accessible by an intermolecular HWE reaction of **36** with **37** using the procedure evaluated above. Final stereoselective CBS reduction and global deprotection liberated archazolid B in 41% yield over 3 steps [42]. This accomplishment presented the second total synthesis of this macrolide while the first total synthesis was enabled by the group of Trauner and will be discussed below.

### Trauner’s retrosynthetic analysis and strategy

**Scheme 8:**
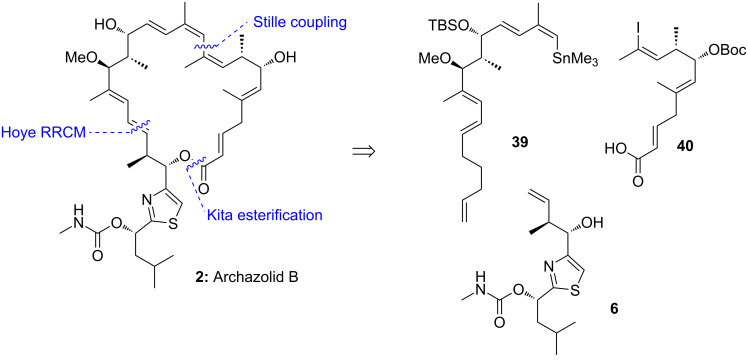
Retrosynthetic analysis of archazolid B by the Trauner group.

Shortly after the total synthesis of archazolid A (**1**) by Menche et al. [41] the total synthesis of archazolid B **2** was reported by Trauner and co-workers [43]. As shown in Scheme 8, they could successfully couple the three main fragments **39**, **40** and **6** by first a Stille reaction, followed by a Kita esterification. Notably, this esterification was critical to avoid unfavorable isomerizations. For closing of the macrolide core they planned a challenging Hoye relay ring closing metathesis (RRCM) which would lead directly after deprotection to archazolid B (**2**).

### Trauner’s total synthesis of archazolid B

#### Synthesis of the north-eastern fragment

**Scheme 9:**
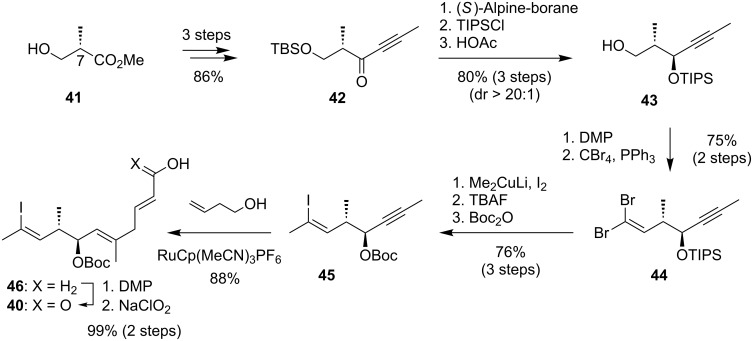
Synthesis of acid **40** from Roche ester **41** involving a highly efficient Trost–Alder ene reaction.

The synthesis of fragment **40** started with the literature-known protocol for generation of ynone **42** derived from (*S*)-Roche ester **41** [79] as shown in Scheme 9. After reduction the alcohol was protected with TIPS and the TBS ether was cleaved by acetic acid to get to the primary alcohol **43**. The reduction with (*S*)-alpine borane was highly diastereoselective (dr > 20:1). Following this sequence over 6 steps the two stereogenic centers at C7 and C8 were successfully built up. The primary alcohol of fragment **43** was then oxidized by the Dess–Martin reagent (DMP) and then treated with CBr_4_ and PPh_3_ to generate the dibromoalkene **44** in high yield of 75% over 2 steps. The group now installed the vinyliodide for the Stille coupling by treating the alkene with lithium dimethylcuprate. In comparison with the likewise attempted Stork–Zhao olefination this protocol by Tanino and Miyashita was superior in yield and stereoselectivity [80]. To complete the fragment synthesis the [Ru]-catalyzed Trost–Alder-ene reaction [81] generated the desired primary alcohol which was oxidized in 2 steps with DMP and NaClO_2_/NaH_2_PO_4_ to the free acid **40**. The high regioselectivity of the Alder-Ene reaction is remarkable and was argued to be derived by a coordinating effect of the carbonate. Also, the overall high yield for synthesis of this elaborate vinyl iodide is impressive.

#### Synthesis of the north-western fragment

**Scheme 10:**
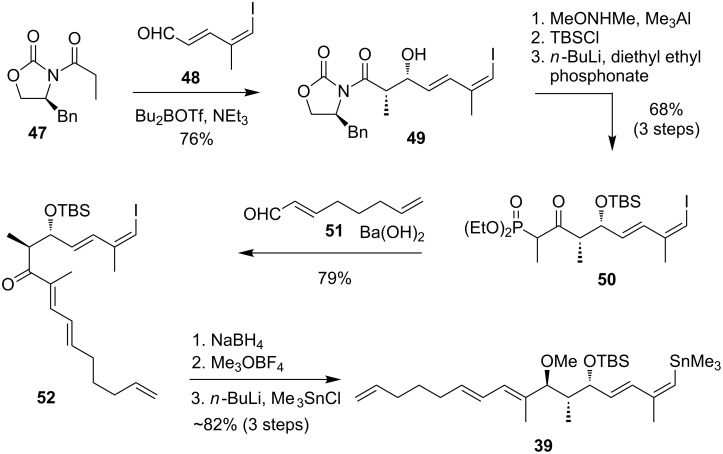
Synthesis of precursor **39** for the projected relay RCM reaction.

As shown in Scheme 10, the synthesis of stannane **39** started with aldehyde **48** which was derived from propargyl alcohol in five steps [82]. After DMP oxidation the generated aldehyde **48** underwent a *syn*-selective Evans aldol addition with oxazolidinone **47** to obtain the alcohol **49** in 76% over 2 steps [83]. Before TBS protection the Weinreb amide was generated and then conversed into the phosphonate **50** as a precursor for a Horner–Wadsworth–Emmons reaction. The olefination led to unsaturated ketone **52** in 79% yield. For the final fragment synthesis the ketone was reduced with sodium borohydride to generate all three required stereogenic centers for this fragment with excellent diasteroselectivity. Final methylation of the free alcohol was followed by conversion of the vinyl iodide into the desired stannane to get fragment **39** which was used directly for coupling.

#### Completion of the total synthesis

**Scheme 11:**
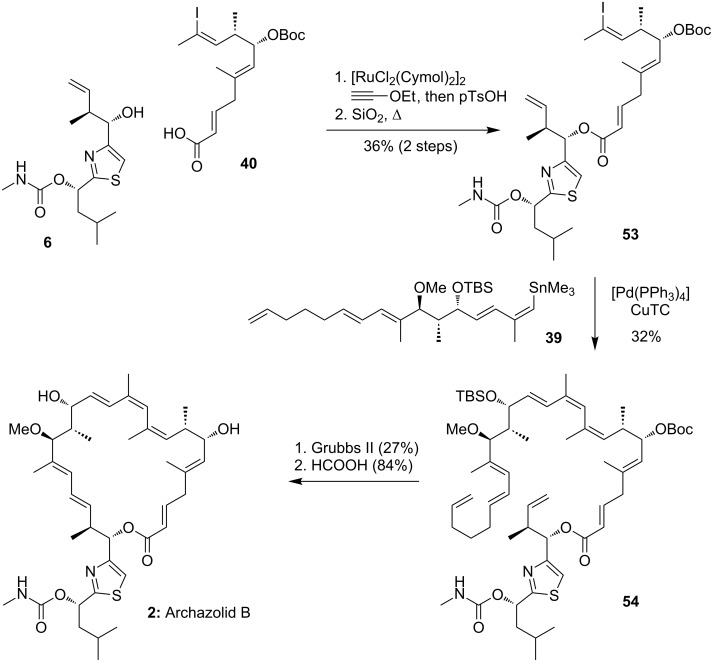
Final steps of Trauner’s total synthesis of archazolid B.

Final assembly of the fragments as shown in Scheme 11 began with an unusual [Ru]-catalyzed Kita esterification due to the instability of fragment **40** towards basic esterification approaches [84]. The following Stille coupling with stannane **39** was then accomplished by CuTC co-catalysis to get the final fragment **54** in 32% yield [85–86]. The envisioned Hoye relay RCM was catalyzed by Grubbs’ second generation catalyst to close the macrocycle in 27% yield. The final acid-mediated deprotection liberated finally archazolid B (**2**). Notably, no cyclization was observed in an analogous RCM reaction with a substrate without the relay tether, which underscores the usefulness of this relay tactic.

It is important to mention, that the three main fragments were coupled in only four steps, showing the highly modular approach from Trauner and co-workers. With this strategy it was possible to synthesize archazolid B (**2**) in only 19 steps from (*S*)-Roche ester **41** (longest linear sequence).

### O'Neil’s retrosynthetic analysis and strategy

**Scheme 12:**
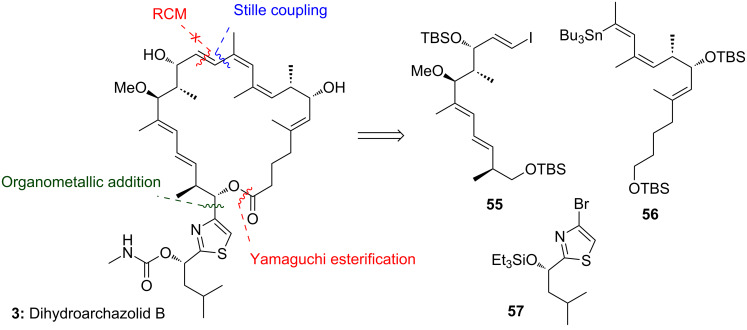
Overview of the different retrosynthetic approaches for the synthesis of dihydroarchazolid B (**3**) reported by the O'Neil group.

As discussed above one of the main difficulties of any archazolid synthesis involves the labile C1 to C5-dienoate system, which is prone to isomerization. However, the similar biological potency of archazolids A and B as well as the archazolid B isomer archazolid F, which bears a 3,4- instead of the 2,3-alkene, suggest that the C2-olefin may not be essential for the biological potency. Accordingly, the group of O‘Neil and co-workers has been targeted dihydroarchazolid B (**3**). They assumed a similar biological potency of the derivative with simultaneous simplification of the synthesis. While so far, they have not been able to finish this synthesis, they have reported several very instructive and efficient fragment syntheses, including the three main fragments **55**, **56** and **57** as shown in Scheme 12 [45–46,48]. The challenging ring-closing metathesis between C13 and C14 could not be established mainly due a competing backbiting process of the corresponding western fragment [46].

### O'Neil’s syntheses of advanced dihydroarchazolid B fragments

#### Synthesis of the macrocyclic skeleton

**Scheme 13:**
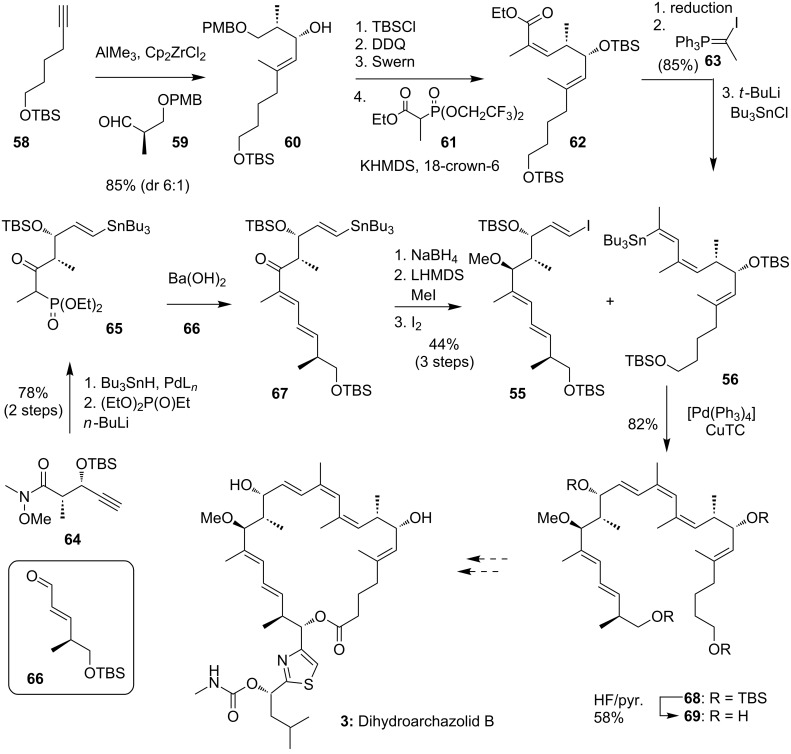
Fragment synthesis of **69** towards the total synthesis of **3**.

Based on this unsuccessful approach O‘Neil and co-workers published a new synthetic route towards 2,3-dihydroarchazolid B (**3**) [48]. As shown in Scheme 13 they were able to synthesize the macrocyclic skeleton **68** by a Stille coupling between stannane **56** and iodide **55** as the key step. Notably, they had to switch the halide/organometallic functionality of each building block after an unsuccessful coupling between the stannane synthesized by reduction and methylation of ketone **67** and the iodine derived from fragment **62**. They assume that the steric hindrance of the methyl group in C10 position possibly lowers the reactivity of the iodine in the oxidative addition step in the catalytic cycle.

For the synthesis of stannane **56** the authors could benefit from the previous fragment synthesis. In 2010 they first published an approach to an eastern building block through an allylation-elimination sequence to form the triene system C9 to C14 [44]. In a second generation fragment synthesis building block **62** was firstly synthesized in 2014 [46]. Starting with the TBS-protected alkyne **58** [87] a zirconium-mediated carboalumination reaction [88] and subsequent coupling with aldehyde **59** gave alcohol **60**. In this Δ^5,6^-*trans*-selective reaction the desired *anti*-diastereomer could be isolated in 85% with a dr of 6:1, which presumably arises from a chelation-controlled stereoselectivity [89]. The aldehyde **59** itself can be prepared in two steps from the corresponding Roche ester [90]. After protection of the free alcohol and deprotection of the primary PMB-protected alcohol with DDQ, the resulting alcohol was oxidized to the corresponding aldehyde. The crude aldehyde was then directly transformed into the (*Z)*-α,β-unsaturated ester **62** as a single stereoisomer by a Still–Gennari [72] olefination with phosphonate **61** in an excellent yield of 93% over 4 steps. After reduction of ester **62** to the corresponding aldehyde (by a method that was not specified by the authors) phosphorane **63** was used to generate the respective *Z*-vinyl iodide in 85% yield as an 8:1 (*Z*,*Z)*:(*Z*,*E)* mixture [91] which was later switched to the stannane **56** by lithium–halogen exchange and further treatment with Bu_3_SnCl [92] in 90% yield.

The synthesis of the coupling partner **55** started with known Weinreb amide **64** which underwent a *syn*-selective palladium-catalyzed hydrostannylation and was then transformed to phosphonate **65** in good yield. For coupling with known aldehyde **66** [93] the O’Neil group chose Ba(OH)_2_ as base for the HWE-type reaction [94] to generate the α,β-unsaturated ketone **67** in 75% yield as a 10:1 mixture of isomers. Similar to the earlier discussed synthesis of Trauner and co-workers [43] reduction with sodium borohydride delivered the desired alcohol in a 10:1 diastereoselectivity. The alcohol was methylated by a protocol involving methyl iodide and LiHMDS, that had been previously used by the group [45]. The stannane was finally converted to the iodide **55** by iododestannylation [95] to complete the fragment synthesis in 44% yield over 3 steps.

For the final step the authors decided to follow a Stille coupling protocol established by Fürstner et al. [96] with CuTC as co-catalyst and [Ph_2_PO_2_][NBu_4_] as tin scavenger. Subsequently, the triene **68** could be synthesized in excellent 82% yield. For biological studies the final fragment **68** was globally deprotected to the alcohol **69**.

To this end, the O’Neil group successfully established a route to the dihydroarchazolid B fragment **68** in only 9 steps (longest linear synthesis). This route also proved that a retrosynthetic disconnection between C12 and C13 can be useful for new approaches to the 24-membered macrolide core. For the completion of the synthesis of **3** the side chain would have to be introduced, followed by an oxidation to the acid and a ring closing esterification.

#### Concise synthesis of the thiazole fragment

**Scheme 14:**
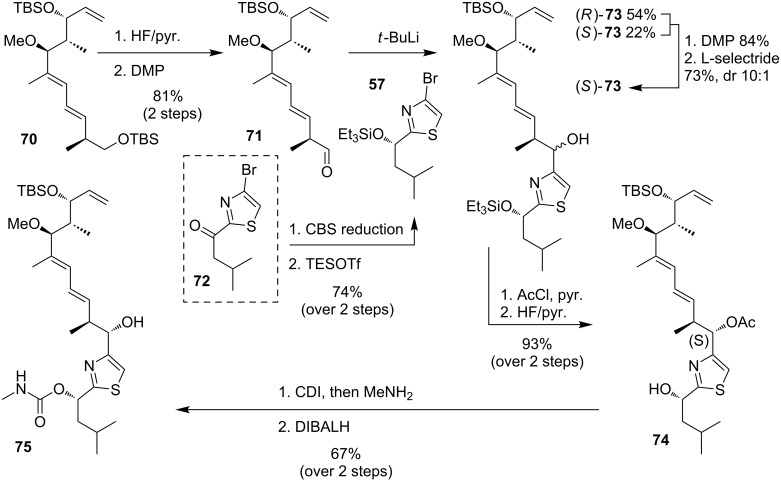
Organometallic addition of the side chain to access free alcohol **75**.

In an earlier synthesis the O’Neil group had already coupled a similar fragment **70** with the southern fragment **57** by an organometallic addition, however, with a lack of stereoselectivity in C23 position [45]. As shown in Scheme 14, they started with the deprotection of the primary TBS ether **70** and DMP oxidation to the aldehyde **71**. The bromide **57**, derived in two steps from literature-known ketone **72** [49], was converted to an organolithium compound which attacked the aldehyde to give the free alcohol **73** in 1:2.5 diasteroselectivity in favor of the undesired *R*-isomer of **73**, which can be explained by the Felkin–Ahn model. For generation of only (*S*)-**73** the both epimers were oxidized to the ketone by DMP followed by reduction with L-selectride. Protection of the free alcohol with acetate and deprotection of the TES group was then required to install the carbamate with CDI and MeNH_2_. After deprotection, fragment **75** was synthesized in 67% yield over 2 steps.

Having these tactics in hand it will be interesting to follow the completion of the first synthesis of dihydroarchazolid B (**3**) by the O’Neil group and the biological data of this compound.

## Conclusion

The discovery of the archazolids led to important advances in the understanding of the role of V-ATPases in cancer development and progression. Based on extensive studies with these macrolides as chemical tools V-ATPases have emerged as a completely novel and highly promising novel class of anticancer targets. Along these lines synthetic chemistry has played a pivotal role, not only by providing these scarce natural products for biological evaluation, but also in supplying novel analogues with tailored functional properties to decipher the target inhibitor interactions at a molecular level. Finally, the total syntheses of the Menche and Trauner group were also of key importance to assign the full stereochemistry in the first place. The various approaches discussed within this manuscript show the various tactics and strategies that may be employed in complex polyketide synthesis. Notable features of the total synthesis by the Menche group include the robustness of boron mediated aldol reactions to set both the characteristic assemblies of neighbored methyl and hydroxy group bearing stereogenic centers. In addition, an aldol condensation was shown to enable an efficient route for construction of a delicate triene system. The final *E*-selective Heck coupling on a highly elaborate substrate and the subsequent HWE macrocyclization are remarkable. The Trauner group in turn effectively employed various ruthenium-catalyzed reactions, including a relay ring-closing metathesis, which demonstrates the powerfulness of such a tactic even for highly elaborate substrates with several initiation positions. However, despite these advances and impressive total syntheses the design and development of a truly reliable and scalable route that will enable an access to gram amounts of thee scarce metabolites continues to present a key scientific challenge and the O’Neil group has already demonstrated that a more concise route may be possible. Efforts are now being directed in the design and development of truly practicable and scalable routes to more stable archazolids to enhance the further preclinical development of these novel anticancer agents. Particular importance will be the development of a truly reliable and high yielding macrocyclization method, while efficient methods for fragment syntheses have been established. It will also be interesting to follow whether synthetic chemists will be successful to establish a scalable route that will enable the synthesis of gram quantities of the authentic natural products or novel archazolids with likewise potent or even improved pharmacological and pharmacokinetic properties to fully exploit the extremely promising biological profile of these polyketide macrolides.
